# Cardiac regeneration in the axolotl is unaffected by alterations in leukocyte numbers induced by lipopolysaccharide and prednisolone

**DOI:** 10.1186/s13104-021-05574-z

**Published:** 2021-04-28

**Authors:** Kathrine Pedersen, Rikke Kongsgaard Rasmussen, Anita Dittrich, Henrik Lauridsen

**Affiliations:** Department of Clinical Medicine (Comparative Medicine Lab), Aarhus University, 8200 Aarhus N, Denmark

**Keywords:** Heart regeneration, Axolotl, Inflammation, Immune system, Lipopolysaccharide, Prednisolone

## Abstract

**Objective:**

Cardiac regeneration in the axolotl has been found to rely on the innate immune system, and especially macrophages have been demonstrated to play a vital role in regulating the regenerative process. In this study we wanted to induce a pro- and anti-inflammatory milieu in the axolotl during heart regeneration to test the resilience of the regenerative response.

**Results:**

This was induced via repeated intrapericardial injections of lipopolysaccharide or prednisolone during a 40-day regeneration period in order to challenge the presumably fine-tuned inflammatory response that normally facilitates regeneration. We observed a local and systemic leucocyte response to pro- and anti-inflammatory stimulation, but we found cardiac regeneration to be structurally and functionally unaffected.

## Introduction

The axolotl is a popular model organism in the field of regenerative medicine, due to its ability to regenerate for instance functional heart muscle after injury [1]. In adult mammals, cardiac injury leads to fibrotic scarring. The response to injury in fetal mammals is dominated by anti-inflammatory macrophages promoting repair, while pro-inflammatory macrophages are more abundant in the adult, causing fibrosis [2]. Even though possible macrophage-polarizations are not well described in the axolotl, macrophages have been shown to be critical for regeneration of cardiac muscle [3] and amputated limbs [4].

We aimed to test the resilience of the pro-regenerative inflammatory response in the axolotl upon challenge with lipopolysaccharide (LPS), prednisolone or saline control. LPS is strongly pro-inflammatory and can induce pro-inflammatory macrophage polarization [5], while prednisolone can inhibit inflammatory macrophage activity and promote anti-inflammatory-polarization in vitro [6, 7].

## Main text

### Methods

#### Animal experiments

Axolotls (weight ± SD = 18.1 ± 3.3 g; total length ± SD = 13.3 ± 0.9 cm) were housed individually at 20 °C on a 12-h light/dark cycle. Animals from a commercial breeder (Exoterra GmbH, Germany) were acclimatized 14 days prior to experimental procedures. All procedures were conducted in dedicated laboratory spaces. General anesthesia was obtained by submersion in benzocaine solution (200 mg l^−1^, 30 min) prior to echocardiography, intrapericardial injections and cryoinfarction surgery. For cryoinfarction surgery, the ventricle was exposed, and a copper probe cooled in liquid nitrogen was applied to the ventroapical face of the heart for 10 s. Incision was closed with individual stiches. Injections were performed by inserting a syringe carrying a 30-gauge needle into the pericardial space. Axolotls were injected every five days starting 5 days prior to cryoinfarction surgery with 20 µl of either sterile amphibian Ringer´s solution (6.6 g l^−1^ NaCl, 0.15 g l^−1^ KCl, 0.15 g l^−1^ CaCl_2_, 0.2 g l^−1^ NaHCO_3_) for saline control, 10 µg of LPS (Sigma Aldrich, L5886) or 20 µg of prednisolone (Sigma Aldrich, P6004), both dissolved in Ringer´s solution. Initial sample size was 12 animals per group. However, due to insufficient knowledge about effect size, power calculations could not be conducted. Forty days post cryoinfarction, animals were euthanized by overdosing animals in benzocaine and harvesting hearts blood and pericardial aspirate.

#### Echocardiography

Starting 5 days prior to injury, animals were imaged with echocardiography every five days up to day 40 (both pre- and post-injury on day 0). Imaging started exactly 30 min after initializing anesthesia and the imaging protocol was identical between animals, due to the cardio-stimulatory effect of benzocaine over time [8]. Animals were imaged in a supine position submerged in water using the Visualsonics Vevo 2100 system and a MS700 (48–50 MHz) transducer as previously described [9]. B-mode recordings (1000 frames, frame rate range of 45–65 f s^−1^) of the ventricle in long-axis view, color-Doppler recordings of the ventricle and pulsed wave Doppler recordings in the outflow tract were obtained for heart rate measurements. Three cardiac cycles were analyzed for each data point using Visualsonics Vevo LAB ultrasound analysis software to measure mid ventricular cross-sectional area at end diastole, end systole as well as mid infarction cross-sectional area and heart rate (HR) to calculate infarction fraction (IF), healed fraction (HF), stroke volume (SV) and cardiac output (CO).

HF of the infarction was calculated at each time point (t) with IF at day 5 as reference:

1 $$HF\left( t \right) = \frac{{IF\left( {day 5} \right) - IF\left( t \right)}}{{IF \left( {day 5} \right)}} \times 100\%$$

SV was calculated from end diastolic area (EDA) and end systolic area (ESA) under the assumption of a spherical ventricle:

2 $$SV = \frac{4}{3} \times \pi \times \sqrt {\frac{EDA}{\pi }}^{3} - \frac{4}{3} \times \pi \times \sqrt {\frac{ESA}{\pi }}^{3}$$

CO was calculated from HR, SV and weight:

3 $$CO = \frac{HR \times SV}{{weight}}$$

#### Collection of ventricle, blood and aspirate

At day 40 animals were anesthetized, and an incision was made into the pericardium to collect pericardial fluid. Pericardial aspirate smears were prepared immediately and fixated in ice cold methanol for 2 min. Axolotls were then injected with 50 µl of 2,500 IU ml^−1^ heparin and the outflow tract was severed to collect arterial blood. Blood smears were prepared in the same way as aspirate smears. The ventricle was harvested, gently rinsed with amphibian Ringer´s solution and transferred to 4% neutral buffered formalin.

#### Histology

Fixated aspirate and blood smears were stained in modified Wright-Giemsa staining solution (Sigma Aldrich, cat. nr: WG128) for 45 s, rinsed in deionized water and mounted with DPX mountant (Sigma Aldrich, cat. nr: 06522). Formalin fixed ventricles were embedded in paraffin and sectioned at 10 µm slice thickness across the entire ventricle, with every fifth section collected and stained using a Masson’s-trichrome kit (Polysciences Inc., cat. nr: 25088) according to the manufacturers protocol. Unbiased stereology was performed as described by Mühlfeld et al. [10] to measure ventricle and infarction volume using a minimum of 12 evenly spaced sections across each heart and counting > 100 point grid intersections with myocardial tissue.

#### Statistical analysis

Relevant t-tests (paired/unpaired) were used to test for statistically significant differences between two groups. Relevant types of ANOVAs (repeated measure, one-way/two-way) were used for omnibus testing of statistically significant difference between more than two groups and evaluate differences between treatment groups and between time points. For post hoc tests of difference between groups, Bonferroni corrected t-tests were used. Significance level (α) = 0.05 unless specified as Bonferroni correction. Unless otherwise specified, reported measurements throughout the text and figures are arithmetic mean ± 95% confidence interval.

### Results and discussion

**Fig. 1:**
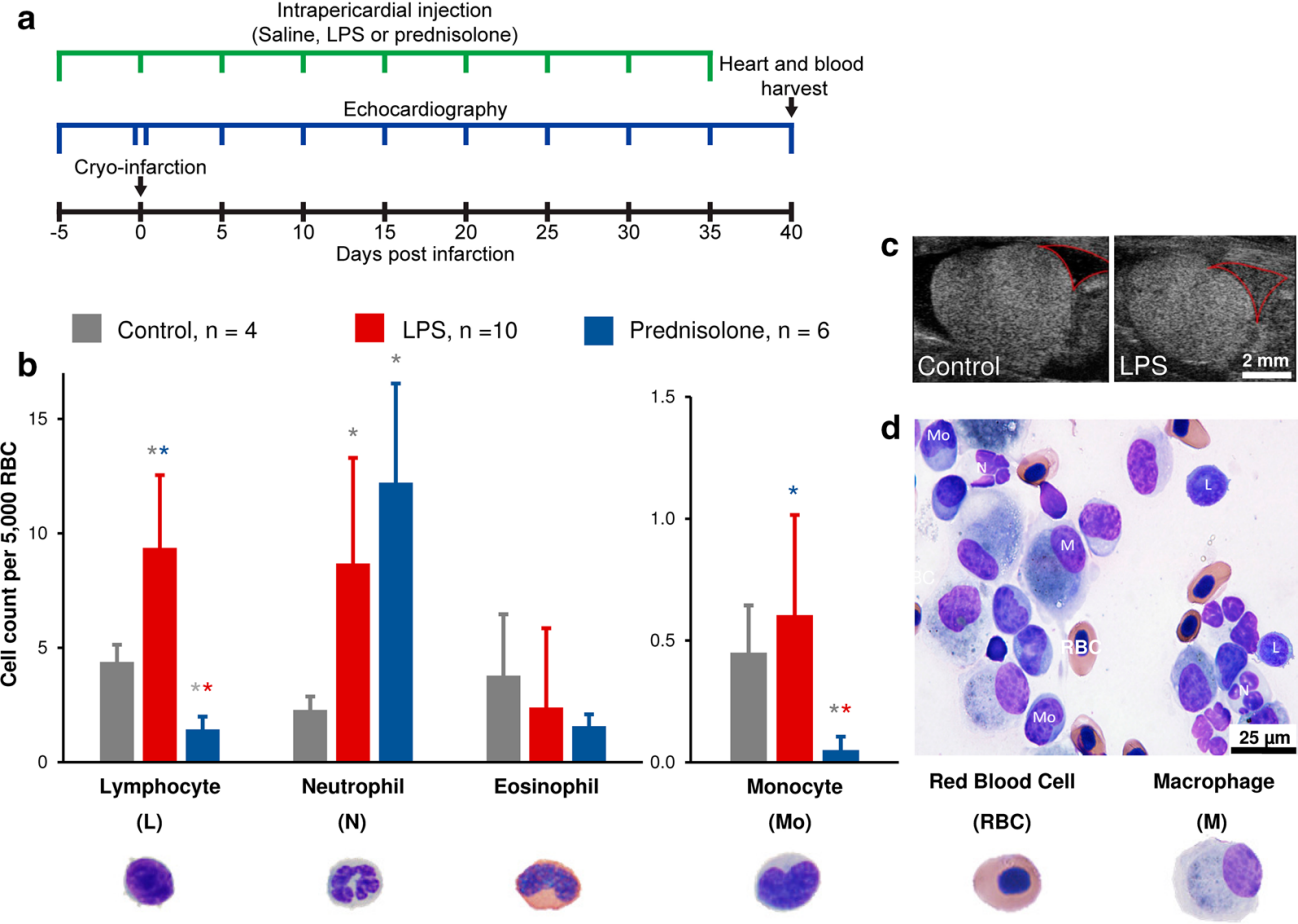
Systemic and local inflammatory response to pro- and anti-inflammatory stimulation. **a** Graphical overview of experiment. **b** Leukocyte numbers expressed as cell count per 5,000 red blood cell (RBC) on Wright–Giemsa stained blood smears. Significant differences shown by asterisk (grey indicating significant difference to control, red to LPS and blue to prednisolone). Representative images of axolotl leukocytes are displayed below each cell category. Error bars represent 95% confidence interval. Two-tailed unpaired students t-test with α = 0.05 was used to determine statistical significance. **c** Representative echocardiography images of control (left) and LPS (right) treated animals with clear pericardial fluid in control and pericardial fluid with nucleated cell infiltrate in LPS (area marked with red line). **d** Representative image of Wright-Giemsa stained smear of pericardial aspirate from LPS treated animal

Continuous injections with either LPS, prednisolone or saline (Fig. 1a), resulted in significant differences between leukocyte counts between treatment groups at day 40 (two-way ANOVA with regression, p = 0.011). Post hoc t-tests showed increased numbers of several types of leukocytes in arterial blood in LPS treated axolotls compared to both control and prednisolone groups (Fig. 1b). The number of lymphocytes and neutrophils were significantly increased in LPS treated compared to control animals (unpaired t-test, lymphocytes: p = 0.014; neutrophils: p = 0.025). Prednisolone treated animals in turn showed significant decrease in numbers of lymphocytes and monocytes compared to control (unpaired t-test, lymphocytes: p < 0.01; monocytes: p < 0.01) but an increase in neutrophil number (unpaired t-test, p < 0.01), reflecting a known effect of prednisolone [11], which does not necessarily indicate increased neutrophil activity. Lymphocyte and monocyte numbers were also significantly higher in LPS compared to prednisolone animals (unpaired t-test, lymphocytes: p < 0.01; monocytes: p < 0.01).

A local inflammatory response in the pericardial space was observed in LPS animals on echocardiography, which showed gradual accumulation of nucleated cells (Fig. 1c). Pericardial fluid aspirated after 40 days of regeneration contained large numbers of leukocytes in the LPS group compared to control and prednisolone groups (Fig. 1d). The lowest level of infiltrate was seen in the prednisolone group. Pericardial aspirate from the LPS group was dominated primarily by macrophages and secondly monocytes, lymphocytes and neutrophils (Fig. 1d).

**Fig. 2:**
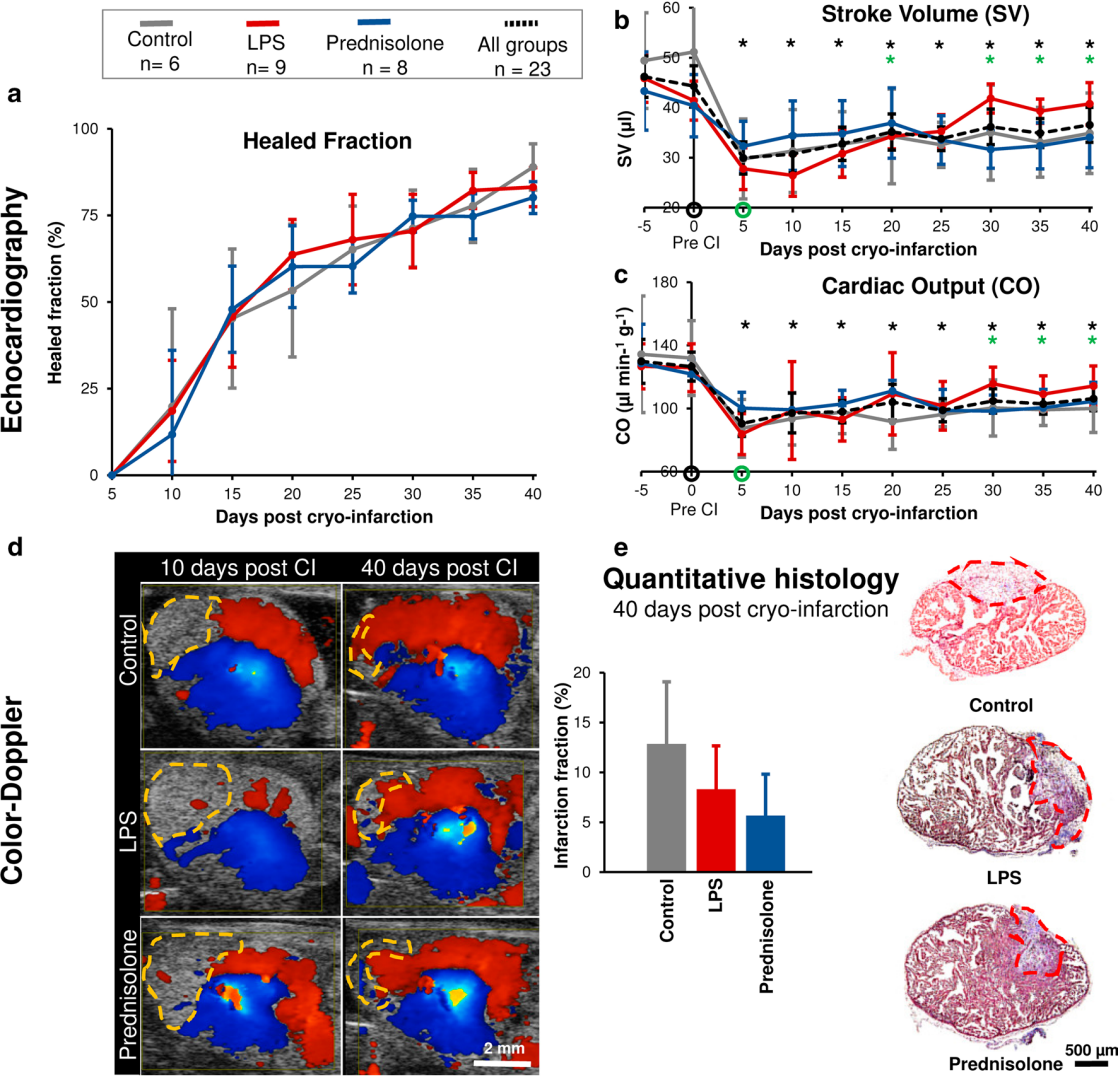
Functional and anatomical cardiac regeneration is unaffected by pro- and anti-inflammatory stimulation. Echocardiography was performed every five days (both pre- and post-infarction on day 0) and hearts were harvested at day 40 for stereology. **a** Healed fraction of infarction relative to maximum infarction size at five days after injury. **b** Stroke volume pre-infarction at day -5 and 0 and thereafter post-infarction. **c** Cardiac output pre-infarction at day -5 and 0 and thereafter post-infarction. Significant differences in combined group shown by asterisks in b and c (black indicating significant difference to day 0 pre-infarction, green to day 5 at maximum infarction). **d** Representative color Doppler images at day 5 and day 40 post injury. Red color indicates blood flow towards transducer (ventral), blue away from transducer (dorsal), and lack of color indicates no blood flow. Infarction area is identified by a lack of blood flow due non-functioning cardiac muscle, and it is highlighted by yellow dashed line. All images were captured at end diastole. **e** Infarction fractions at day 40 measured by quantitative histology using unbiased stereology and representative images of Masson’s-trichrome stained hearts at day 40 from control, LPS and prednisolone animals with remaining infarction indicated by red dashed line. Error bars on all panels represent 95% confidence interval

The level of regeneration and cardiac function was assayed longitudinally by echocardiography (Figs. 1a, 2a–d). Structural regeneration was evaluated by the size of the infarction zone measured as ventricle volume not contracting. Immediately after cryoinjury, mean infarction fraction constituted 8.6 ± 1.4% of ventricle volume across all groups (n = 24). Infarction fraction significantly increased (paired t-test, n = 24, p < 0.01) to constitute 18.3 ± 4.3% of ventricle volume five days after cryoinjury where the injury zone was fully developed. No significant difference was observed in fold change of infarction fraction between groups (One-way ANOVA, p = 0.74). To assess anatomical regeneration, maximum infarction size at five days after injury was used as reference to measure subsequent healed fraction until 40 days after injury (Fig. 2a). The infarction zone was gradually regenerated (Fig. 2a) (Two-way ANOVA with repeated measures, p < 0.01 with Bonferroni corrected (α/7 = 0.0071) post hoc t-tests showing significant increased healed fraction in all groups from 15 days until 40 days post injury). No significant difference was observed in healed fraction between groups at any time point (Two-way ANOVA with repeated measures, p = 0.96). 40 days after injury, the healed fraction was 83.8 ± 3.4% across groups (n = 23).

Quantitative histology using unbiased stereology, measuring infarction fraction on Masson’s trichrome-stained sections across entire hearts harvested 40 days post injury showed no significant difference in remaining infarction fraction (One-way ANOVA, p = 0.17) (Fig. 2e) or ventricular myocardial volume between groups (One-way ANOVA, p = 0.58). Across groups ventricular myocardial volume was 2.47 ± 0.33 µl 40 days after injury and the remaining infarction fraction was 8.45 ± 2.90%.

Stroke volume and cardiac output was used to evaluate functional recovery after cryoinjury. Cryoinjury significantly affected cardiac function for both stroke volume (Two-way ANOVA with repeated measures, p < 0.01) and cardiac output (Two-way ANOVA with repeated measures, p < 0.01). However, functional regeneration progressed similarly with no significant difference between groups for both stroke volume (Two-way ANOVA with repeated measures, p = 0.98) and cardiac output (Two-way ANOVA with repeated measures, p = 0.54). Five days after injury stroke volume and cardiac output across all groups was significantly reduced to 69.0 ± 6.4% and 72.7 ± 6.6%, respectively, compared to immediately before injury (Paired t-tests, n = 24; Stroke volume: p < 0.001; Cardiac output: p < 0.01). Thirty days after cryoinjury stroke volume and cardiac output were significantly increased compared to minimum function at 5 days after injury (Bonferroni corrected (α/7 = 0.0071) post hoc t-tests) (Fig. 2b, c). This demonstrated functional recovery, although not to the initial level since stroke volume and cardiac output were significantly lower at 40 days after injury compared to pre-injury; 82.5 ± 10.0% and 87.4 ± 6.8% respectively (Paired t-tests, n = 23; Stroke volume: p < 0.01; Cardiac output: p < 0.01) (Fig. 2b, c). Imaging the heart using color Doppler imaging, qualitatively showed compromised blood flow in the infarcted area in early time points after injury, which was close to normal after 40 days of regeneration with no difference between groups (Fig. 2d).

Taken together, our results strongly suggests that although there are alterations in local and systemic leukocyte numbers in response to LPS and prednisolone, it does not affect cardiac function nor the rate or fidelity of cardiac regeneration following injury in the axolotl compared to control animals. As previously demonstrated, LPS stimulation does alter cytokine profiles in the axolotl [11], thus supporting that the altered leukocyte counts found here in response to LPS injections represents an altered inflammatory cardiac environment.

With a previous demonstration of the importance of macrophage function in axolotl cardiac regeneration [3], it is somewhat surprising that structural and functional cardiac regeneration is unaffected by significant alterations in leukocyte numbers. Polarization of macrophages in vivo is believed to be on a spectrum, rather than the M1/M2 phenotypes described in vitro [12], however, it remains central to the understanding of macrophage phenotypes.

One hypothesis of how the immune response is more favorable towards regeneration in fetal and neonatal mammals as well as regenerative model organisms like the zebrafish and the axolotl is that the more naïve immunological state in these organisms falls short of mounting a substantial pro-inflammatory response to injury with the benefit of reduced fibrosis and increased ability for regeneration as reviewed by Sattler and Rosenthal [13]. This does, however, seem somewhat counterintuitive to the massive increase in leukocyte numbers observed in response to LPS in the current study, with cardiac regeneration proceeding unaffected. It may also be indicative of critical differences in the axolotl versus mammalian immune function with respect to regeneration. A comparative study of the cardiac regeneration competent zebrafish and the cardiac regeneration incompetent medaka, brings the idea of a low-level immune response being favorable for regeneration further into questioning. It was shown that the regenerating zebrafish compared to the medaka, in fact mount a strong inflammatory response more quickly with an increased level of macrophage infiltration, which in turn cleared more efficiently [14].

Deducing the exact phenotype of macrophages involved in cardiac regeneration in the axolotl will be highly relevant to further the understanding of the regenerative process and the knowledge may one day be translated into human therapies. The tolerance of the macrophage dependent regenerative response to LPS and prednisolone indicate that the reparative phenotype is not subject to polarization towards a non-reparative pro-inflammatory -type via LPS/toll-like receptor signaling or anti-inflammatory inactivation by corticosteroid/prednisolone signaling, although these clearly have an effect on leukocyte numbers both locally and systemically.

## Limitations

A valuable sophistication of the experiment would be repeated blood sampling during the regenerative process in the axolotl groups to allow for leukocyte counting and cytokine profiling throughout the regenerative process. This would however require larger animals to be ethically acceptable.

## Data Availability

The datasets used and/or analyzed during the current study are available from the corresponding author (HL) on reasonable request.

## Abbreviations

LPS: Lipopolysaccharide
SD: Standard deviation
B-mode: Bright mode
HR: Heart rate
IF: Infarction fraction
HF: Healed fraction
SV: Stroke volume
CO: Cardiac output
t: Time
EDA: End diastolic area
ESA: End systolic area
BM: Body mass
